# Demographics of malignant melanoma in Trinidad and Tobago

**DOI:** 10.1308/rcsann.2026.0012

**Published:** 2026-04-16

**Authors:** MJ Ramdass, J Rattan, A Ribeiro, M Johncilla, S Sandy, S Barrow

**Affiliations:** ^1^ Port of Spain General Hospital, Trinidad & Tobago; ^2^ University of the West Indies, St. Augustine Campus, Trinidad & Tobago; ^3^ Royal College of Surgeons of Ireland, Ireland

**Keywords:** Malignant melanoma, West Indies, Caribbean, Trinidad

## Abstract

**Introduction:**

This study aims to describe the demographics of malignant melanoma (MM) in Trinidad and Tobago.

**Methods:**

All cases of MM for the period 2004–2019 were analysed with data collected on age, gender, histological subtype, anatomical location, Clark Staging, Breslow thickness/American Joint Committee on Cancer 8 guidelines.

**Results:**

There were 54 cases comprising 31 (57%) women and 23 (43%) men with an age range of 12–96 years (mean=61). Incidence was calculated at 0.482 per 100,000 persons per annum and prevalence was 0.01%. The number of new cases diagnosed annually ranged between two and four, with a range of one to seven. African ethnicity was predominant (45; 83.3%) followed by East Indians (6; 11.1%) and mixed (3; 5.6%). Anatomical sites (*n*=51) included the lower limb (28; 54.9%), trunk (9; 17.6%), upper limb (6; 11.7%), ocular region (5; 9.8%), head and neck (1; 2%), head and neck mucosal (1; 2%) and gastrointestinal mucosal (1; 2%). Common subtypes (*n*=42) included not otherwise specified (27; 64.3%), nodular melanoma (NM) (6; 14.3%), acral lentiginous melanoma (ALM) (4; 9.5%), spindle cell (3; 7.1%), superficial spreading melanoma (SSM) (1; 2.4%), amelanotic melanoma (AM) (1; 2.4%). Breslow thickness and the Clark classification were used for 23 and 16 patients, respectively.

**Conclusions:**

There were 54 cases diagnosed over 16 years serving a catchment of 700,000 persons giving an incidence rate of 0.482 per 100,000 persons per annum and a prevalence of 0.01%. African ethnicity was dominant (83.3%) with the lower limb, trunk and upper limb being most commonly affected. There is a major need for better recording of data, staging and treating this disease in the Caribbean region.

## Introduction

Malignant melanoma (MM) is the fifth most common cancer in the United States and is most prevalent among people of White ethnicity, accounting for 98% of cutaneous melanomas.^1^ Although it represents less than 5% of diagnosed skin cancers, MM is responsible for more than 70% of skin-cancer-related deaths, with an increasing incidence of 3% annually for the last 25 years.^2^ In the UK there are 17,500 new cases diagnosed annually, with it being the fifth most common cancer and accounting for 5% of all new cancer cases; incidence rates are highest in the 85- to 89-year-old age group.^3^ It is caused by the malignant transformation of melanocytes, which arise from the neural crest cells and migrate to the basal layer of the epidermis. They can also be found in the leptomeninges, uvea and mucus membranes.^4,5^ The traditional classifications related to prognosis include those by Wallace Clark in the 1960s^6,7^ followed by Breslow in the 1970s.^8^ This is now further standardised by the American Joint Committee on Cancer (AJCC) (version 8, implemented in 2018) guidelines, which describe the condition by depth of invasion, presence of lymph node involvement and metastases^9,10^ and is now the accepted method of classification as it relates to prognosis worldwide. Of note, the presence of ulceration is an adverse prognostic factor and it is likely that the extent of mitotic activity will also be included in the next version of the AJCC.^11^

Melanoma cases are increasing notably worldwide. Prognosis depends on the stage and can differ between ethnicities. Trinidad and Tobago has a population of 1.4 million with a diverse ethnic composition. This study serves as a baseline analysis of the epidemiology of the disease in this country.

## Patients and methods

A retrospective analysis was conducted on the medical records of all MM cases presenting for the 16-year period 2004–2019 at the Port-of-Spain General Hospital, which has a population catchment of 700,000. Patient notes were obtained based on the electronic database at the Department of Pathology. The available histology slides were reviewed by an independent pathologist to confirm the diagnosis. Patients were anonymised and, using a data sheet, information was collected on age, gender, ethnicity, anatomic location, histological subtype, Breslow thickness and Clark Staging. The stage was converted to the format of the AJCC version 8 guideline with the histology available.

Ethnicity was defined as follows: African descent, East Indian and Mixed. In subtypes, NOS refers to not otherwise specified. Data were then recorded and analysed using SPSS version 24 and Chi-square tests were applied with regard to ethnicity and the various variables. Ethics board approval was obtained from the relevant authority and the need to obtain informed consent was waived as this was a retrospective study with no patient identifiers.

## Results

There were 54 cases of MM, comprising 31 (57.4%) women and 23 (42.6%) men with an age range of 12–96 years (mean=61). There were 45 (83.3%) persons of African descent followed by 6 (11.1%) of East Indian descent and 3 (5.6%) were classified as Mixed. The main age groups affected included the 51- to 60-year-old and 61- to 70-year-old groups, accounting for 55.6% of the dataset (Figure 1).

**Figure 1:**
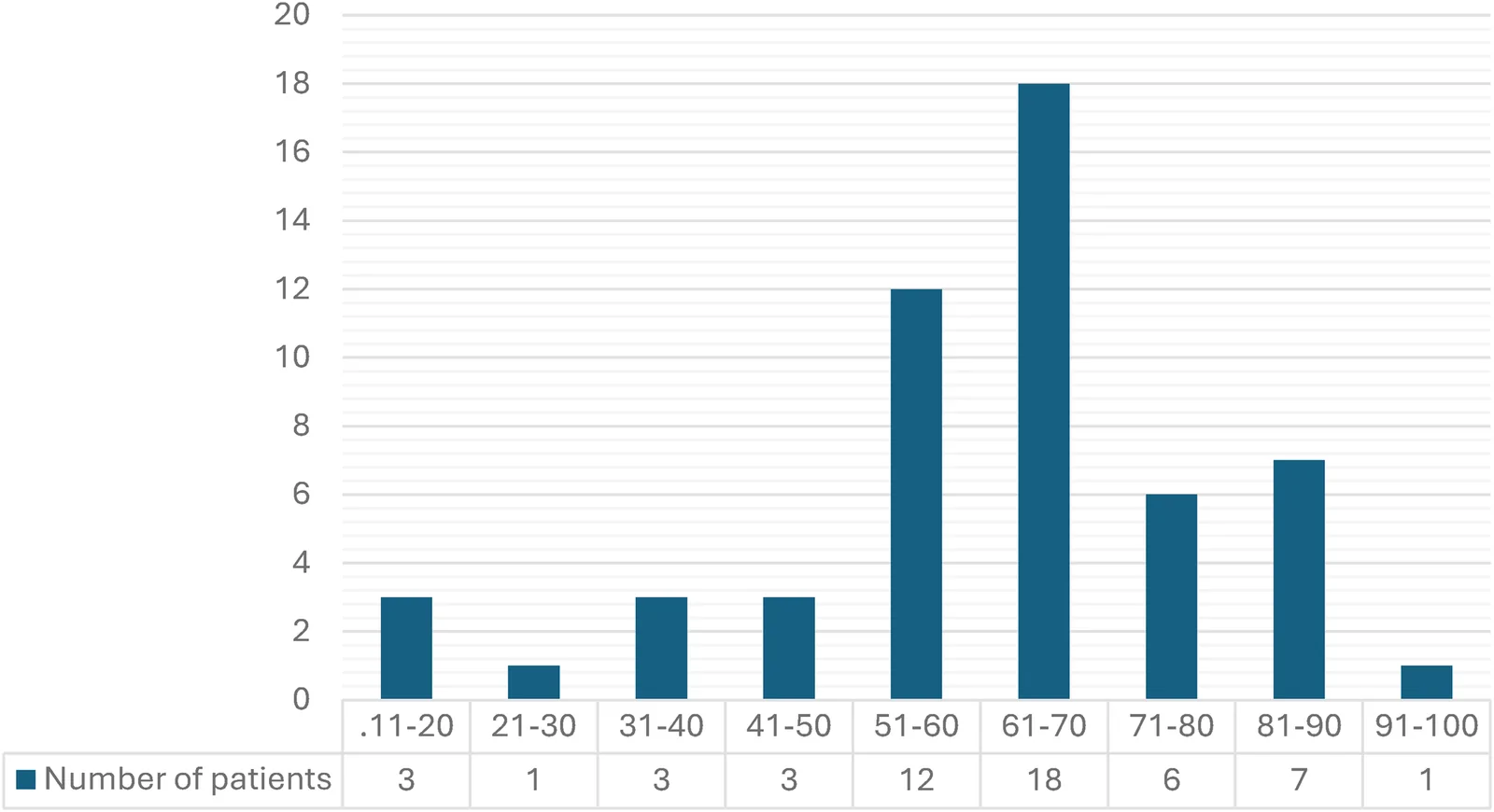
Age distribution of 54 melanomas over 16 years (2004–2019)

Incidence was calculated at 0.482 per 100,000 persons per annum, with 54 cases over 16 years in this catchment in a population consisting of 700,000. The prevalence was calculated at 0.01%. The number of new cases diagnosed annually ranged between two and four with a range of one to seven (Figure 2).

**Figure 2:**
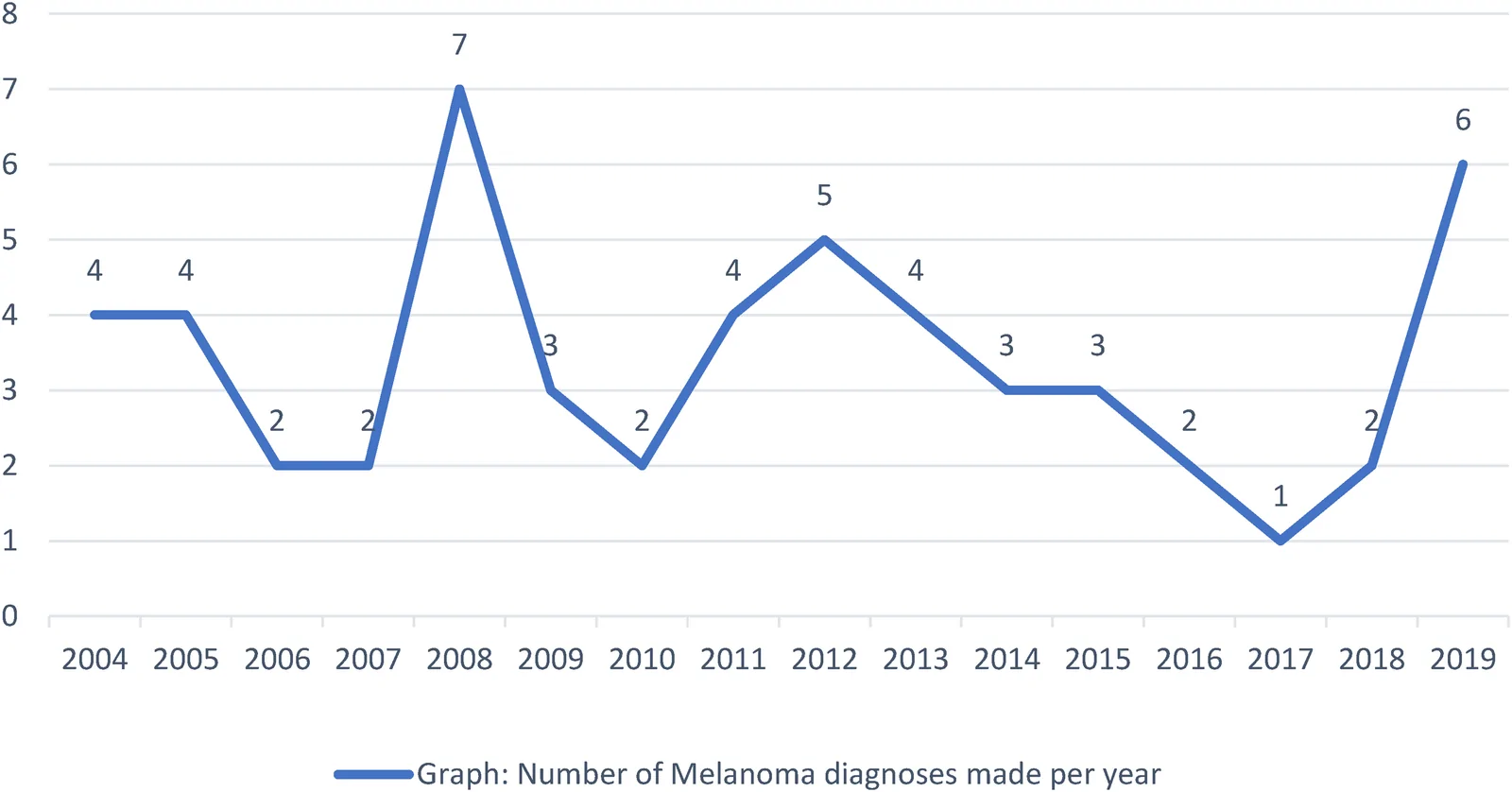
Number of new cases of melanoma diagnosed annually (*n*=54)

Data were missing for three cases (5.6%) with regards to the anatomical location, giving a dataset of *n*=51 with the majority affecting the lower limb (28 cases; 54.9%), the trunk (9 cases; 17.6%), the upper limb (6 cases; 11.7%), ocular region (5 cases; 9.8%) followed by head and neck (1 case; 2%), head and neck mucosa (1 case; 2%) and finally gastrointestinal mucosal (1 case; 2%) (Table 1 and Figure 3). Of note, four out of the five ocular melanomas were in the Afro-Caribbean subgroup.

**Figure 3:**
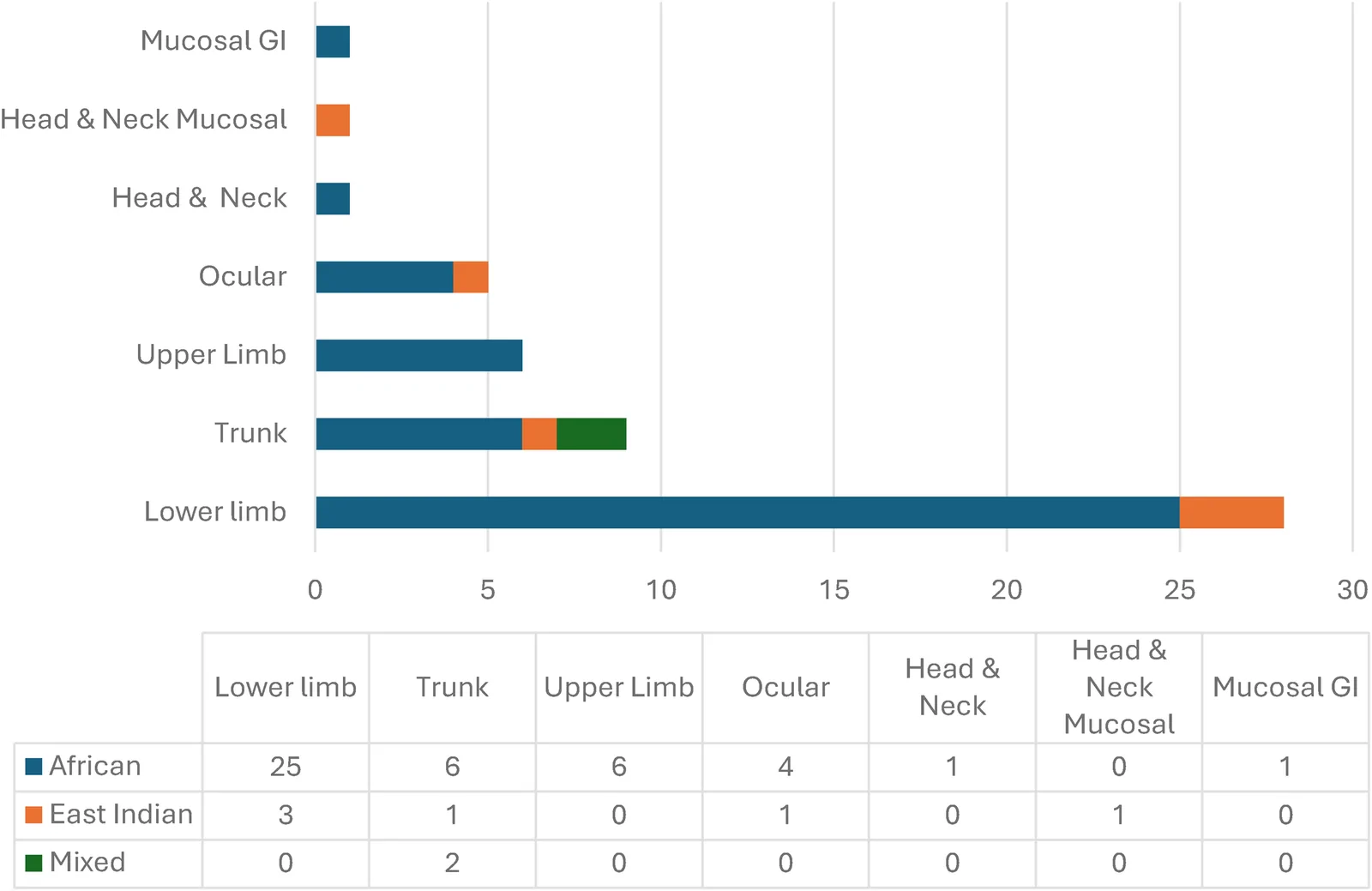
Anatomical distribution of melanomas (*n*=51)

**Table 1:** Demographic data of MM from the Port of Spain general hospital in Trinidad over the period 2004–2019.

	Total *n*=54	Ethnicity		
		African, *n*=45	East Indian, *n*=6	Mixed, *n*=3
Age at diagnosis (years)				
Mean	61	61.8	60.2	66.7
Range	12–96	12–96	32–76	59–82
Sex				
Male	23 (42.6%)	19	4	–
Female	31 (57.4%)	26	2	3
Anatomical location (data missing in 3 of 54 cases)	*n*=51	*n*=43	*n*=6	*n*=2
Lower limb	28 (54.9%)	25	3	–
Upper limb	6 (11.7%)	6	–	–
Head and neck	1 (2%)	1	–	–
Head and neck mucosal	1 (2%)	–	1	–
Trunk	9 (17.6%)	6	1	2
Ocular	5 (9.8%)	4	1	–
Gastrointestinal mucosal	1 (2%)	1	–	–
Histologic subtypes (data missing in 12 of 54 cases)	*n*=42	*n*=34	*n*=6	*n*=2
Lentiginous	4 (9.5%)	4	–	–
Nodular	6 (14.3%)	6	–	–
Superficial spreading	1 (2.4%)	1	–	–
Amelanotic	1 (2.4%)	1	–	–
Spindle cell	3 (7.1%)	–	2	1
NOS (data missing for 15 of 54 cases)	27 (64.3%)	22	4	1
Breslow depth/thickness (AJCC 8 guidelines)	*n*=23	*n*=18	*n*=4	*n*=1
I (≤1.0mm)	2	2	–	–
II (>1.0–2.0mm)	7	6	1	–
III (>2.0–4.0mm)	2	2	–	–
IV (>4.0mm)	12	8	3	1
Clark staging (invasion into)	*n*=16	*n*=14	*n*=2	*n*=0
I (melanoma in situ)	1	1	–	–
II (papillary dermis)	1	1	–	–
III (junction papillary and reticular dermis	2	2	–	–
IV (reticular dermis)	6	5	1	–
V (subcutaneous fat)	6	5	1	–

AJCC = American Joint Committee on Cancer; MM = malignant melanoma; NOS = not otherwise specified

Regarding histologic subtypes, data were missing for 12 cases (22.2%) giving a dataset of *n*=42 with the most common being NOS (27; 64.3%), NM (6; 14.3%), ALM (4; 9.5%), spindle cell (3; 7.1%), SSM (1; 2.4%) and AM (1; 2.4%) (Table 1).

Breslow depth/thickness was available for 23 cases (42.6%) and Clark Staging for 16 cases (29.6%) with data missing for 15 cases (27.8%) (Table 1). T-stage and lymph node metastases were poorly recorded and were excluded from the analysis.

## Discussion

The global incidence and mortality of MM are 3.3 and 0.55, respectively, per 100,000 population worldwide in 2020, with Australia and New Zealand having the highest rates of 33.6 per 100,000 and 33.3 per 100,000, respectively. Risk factors include smoking, alcohol consumption, unhealthy diet, obesity and metabolic diseases, and age 50 years and above in both sexes have an increasing incidence.^12^ Within Europe, the Scandinavian countries have the highest incidence and the Mediterranean countries have the lowest incidences. This can be explained by keeping in mind that two of the biggest risk factors for melanoma are ambient ultraviolet light exposure and fair skin. Countries closer to the equator are noted to have the highest rates of melanoma but there is a distinct decrease in the Mediterranean countries because of their darker skin. African and Asian populations have a low incidence due to their darker pigmentation.^12^

In Trinidad and Tobago, MM is uncommon, as demonstrated by the data for the period 1995–2009, during which lower limb melanomas accounted for 0.11% of cancers in Trinidad and Tobago.^13^ In our study, we had 54 cases of MM diagnosed over 16 years in a catchment serving 700,000, persons giving an incidence rate of 0.482 per 100,000 persons per annum and a prevalence rate of 0.01% for the study period, which is in keeping with people of darker skin.

In Jamaica, a review of cutaneous melanoma (CM) revealed a total of 220 cases diagnosed over 50 years, with an average incidence of 0.9 per 100,000 persons per year.^14^ Martinique – a French West Indian island with a predominantly Black population – had an incidence of 1.48 per 100,000 per year. This low incidence is in keeping with the rates among the African-American population.^15^

In this study we found that melanomas were more common in women than in men (57.4% vs 42.6%). This was further reflected in our subset analyses, where our Afro-Caribbean patients had a male to female (M:F) ratio of 1:1.4 and our East Indian patients had an M:F ratio of 2:1. All three patients in our Mixed group were female. This female dominance is in keeping with regional data from Jamaica and Martinique. However, Butler found the reverse to be true in his analysis of melanoma cases over a 12-year period in which 23 men and 16 women were identified in 1967.^16^ In international data, the sex distribution was found to vary with a male preponderance (2:1) in the United States and Australia. Norval *et al* found that, in Black South Africans, incidence of cutaneous melanoma was slightly higher in women.^17^

In our study, the age range was 12–96 years, with a mean age of 61, which is comparable with other islands in the Caribbean. In the literature, melanoma was most commonly diagnosed in the sixth decade of life but Lodder *et al* described a wider distribution between the sixth and seventh decades^18^ and this was observed in both White and non-White populations.

Comparing ethnicities, it was noted that the East Indian group in this study presented at the youngest mean age at 60.2 years, the African group at 61.8 years and the Mixed race group at 66.7 years.

Anatomical subtypes were as follows: SSM, NM, ALM, AM and NOS. There were three rare cases of spindle cell subtypes. In this study, 14.3% (*n*=6) of patients had NM, all of which were in the African descent group (Table 1).

ALM is very uncommon (<5%), and is seen mainly in darker people on the palms of the hands, soles of the feet and subungually. It is associated with a poor prognosis. Recent studies suggest that ALM has unique patterns of genetic mutations and higher Breslow thicknesses compared with other types of CM, which may contribute to its aggressive nature.^19^ In a Korean study, Ho Joo Jung *et al* demonstrated that melanoma on non-sun-exposed sites of the body may be due to the lesion being on a weightbearing site.^20^ Phan *et al* found that a history of trauma was associated with more advanced disease on the hands and feet; however, most of their patients were White.^21^ There were no studies associating trauma or weightbearing site lesions with ALM in people of Black ethnicity. The majority of our patients (54.9%) had melanomas affecting the lower limb. This is comparable with studies originating in Jamaica, where more than 60% of patients had lesions in the lower limb, in particular the soles of the feet. In White populations, the trunk is affected more in men and the lower limb is affected more in women.^16^

The spindle cell type of CM is rare and accounts for 3–14% of all melanomas. It is predominantly found in White men and usually occurs in the sixth to eighth decade of life. The main sites of these tumours are in the skin, eye or bony orbits.^22^ Spindle cell melanoma made up 7.1% of melanomas in this study.

Over the 16-year interval, it was noted that 49% of the cases did not have a stage in the final histology report but seemed to have an advanced stage when diagnosed. This may be due to a lack of awareness by patients, who may ignore these lesions in the early stages or attempt to self-treat using herbal remedies, religious practices and/or special natural dressings, which is a cultural practice in the Caribbean basin.

Trinidad and Tobago is a multi-ethnic country that comprises primarily East Indians (40.3%), Africans (40%) and Mixed races (14%), with the remainder being European White, Spanish, Chinese and Arabic. It was notable that 83.3% (*n*=45) of all patients were of Afro-Caribbean descent, with 11.1% (*n*=6) being East Indian. This may be due to the catchment where the Port-of-Spain General Hospital is located and can be interpreted as a bias.

There was a lack of standardisation in pathological reporting, with many reports missing important prognostic information; this may be due to a change at the histopathologist level of reporting over the timeperiod of the study.

## Conclusion

In conclusion, this study demonstrates an African descent predominance in patients presenting with MM in Trinidad, with generally more advanced disease in the lower limbs, upper limbs, truncal and ocular structures than other ethnicities. There is a need for better awareness of pigmented lesions in patients of African descent in our population, and to carefully manage and treat these patients with standardised international protocols. We recommend that there should be increased awareness among our population and our primary care physicians to have a low threshold of suspicion in particular for the acral and subungual variants.

## Competing interests

The author/s declare no competing interests.

## Funding

The author/s received no financial support for the research, authorship and/or publication of this article.

## Ethics approval and consent to participate

Ethics approval was granted from the local ethics board of the North West Regional Health Authority.

## Author contributions

**M Ramdass:** Conceptualization, Data curation, Formal analysis, Methodology, Project administration, Writing – original draft, Writing – review & editing. **J Rattan:** Data curation, Formal analysis, Writing – review & editing. **A Ribeiro:** Conceptualization, Data curation, Formal analysis, Project administration, Writing – original draft, Writing– review & editing. **M Johncilla:** Formal analysis, Resources. **S Sandy:** Data curation, Formal analysis, Resources, Supervision. **S Barrow:** Conceptualization, Data curation, Project administration, Resources.

## Artificial Intelligence

The author/s declare that no AI was used to conduct the study or prepare the manuscript.
